# Accuracy of Self-Reported Physical Activity Levels in Obese Adolescents

**DOI:** 10.1155/2014/808659

**Published:** 2014-08-26

**Authors:** Sarah A. Elliott, Kimberley A. Baxter, Peter S. W. Davies, Helen Truby

**Affiliations:** ^1^Children's Nutrition Research Centre, Queensland Children's Medical Research Institute, Royal Children's Hospital, The University of Queensland, Herston, QLD 4029, Australia; ^2^Department of Nutrition and Dietetics, Faculty of Medicine, Nursing and Health Sciences, Monash University, Notting Hill, VIC 3168, Australia

## Abstract

*Introduction.* Self-reported measures of habitual physical activity rely completely on the respondent's ability to provide accurate information on their own physical activity behaviours. Our aim was to investigate if obese adolescents could accurately report their physical activity levels (PAL) using self-reported diaries.* Methods.* Total energy expenditure (TEE) was measured using doubly labelled water (DLW) and resting energy expenditure (REE) was measured via indirect calorimetry. Activity energy expenditure (AEE) and PAL values were derived from measured TEE and REE. Self-reported, four-day activity diaries were used to calculate daily MET values and averaged to give an estimated PAL value (ePAL).* Results.* Twenty-two obese adolescents, mean age 13.2 ± 1.8 years, mean BMI 31.3 ± 4.6 kg/m^2^, completed the study. No significant differences between mean measured and estimated PAL values were observed (1.37 ± 0.13 versus 1.40 ± 0.34, *P* = 0.74). Bland Altman analysis illustrated a significant relationship (*r* = −0.76, *P* < 0.05) between the two methods; thus the bias was not consistent across a range of physical activity levels, with the more inactive overreporting their physical activity.* Conclusion.* At an individual level, obese adolescents are unlikely to be able to provide an accurate estimation of their own activity.

## 1. Introduction

The accurate and reliable assessment of physical activity among children and adolescents is important for many reasons. Physical activity is often assessed in large population based studies to detect group changes in physical activity levels, estimate population prevalence and trends, and evaluate the efficacy of interventions which aim to alter physical activity levels. The assessment of physical activity can also be used to guide dietetic advice, by assisting with the calculation of individual energy requirements.

Numerous methods exist for the assessment of physical activity. Broadly, the various techniques can be grouped as self-report, observation, heart rate monitoring (HRM), motion sensors, and doubly labelled water (DLW), which measures total energy expenditure (TEE) over time [1]. While the DLW technique is considered the most accurate method available for the assessment of daily TEE, which includes energy spent in physical activity in free-living subjects, however, its high cost and demanding methodology render the DLW method unavailable for routine clinical use. Additionally, other techniques, such as HRM and motion sensors, also have methodological drawbacks [1]. Consequently, often pragmatic considerations such as cost and participant burden lead to self-report as the tool of choice. Diaries represent a low cost and time efficient way to record physical activity patterns, timing, and relative intensity of physical activity, compared with criterion measures [2].

Self-reported questionnaires are useful in large-scale studies, but they rely on the child (or parent) to recall physical activity behaviour information accurately. Numerous self-reported instruments are available, with several being used in children. The four major types are (i) self-administered recall, (ii) interview-administered recall, (iii) diaries, and (iiii) proxy reports [3].

While some studies have reported a 73.4% to 86.3% agreement between these instruments and direct observation, these techniques should be used cautiously in pediatric populations in which recalling such information may be difficult. More recent evidence of the reliability and validity of physical activity assessments in children has been examined by Kohl III et al. [4], who reported a low to moderate validity for self-reported measures of physical activity.

Evidence on the use of self-reported measures of physical activity in health children is contradictory. Sallis et al. [5] in 1993, suggested that physical activity recalls of children as young as the fifth grade are of adequate reliability and validity to use in research on physical activity in children. However, Arvidsson et al. [6] have documented that, in comparison to DLW measures of physical activity, the physical activity questionnaire for adolescents was not able to accurately predict physical activity in a group of Swedish adolescents. As a result of this conflicting evidence, Corder et al. [7], just recently, assessed the validity and reliability of four self-reports to assess physical activity and time spent at moderate and vigorous intensity physical activity in British young people between 4 and 17 years of age, against measures of DLW. The strength of association between questionnaire and criterion methods varied (*r* = 0.09 to *r* = 0.46). While some questionnaires were able to accurately assess group-level physical activity, the error was large for individual-level estimates.

While there have been a variety of studies investigating the use of self-reported measure of physical activity in healthy children and adolescents, little evidence of the use of such tools is available in obese children and adolescents. Dated studies by Heitmann and Lissner [8] and Waxman and Stunkard [9] reported that obese boys were less active than nonobese boys at home, but there were no differences in their activities when they were in a playground. It was also suggested that self-recorded activity records suffered from the same potential compliance problems as self-reported dietary intake and thus should be compared with more direct measures of activity [4, 10].

The accuracy of physical activity diaries, as stated by Sallis et al. [5], is highly dependent on the cooperation of the subject. In healthy children and adults, diary based self-reported instruments can provide, with good participation compliance, accurate assessments of habitual physical activity level (PAL) values [11]. However, self-reported measures of activity are fraught with difficulties in obese adult populations, who tend to overreport activity and underreport dietary energy intake [12]. It is unknown if these reporting difficulties are also found in obese children and adolescents. Thus, the aim of this study was to assess how accurately obese children and adolescents self-report their PAL values and to quantify the energy expended during physical activity.

## 2. Methods

Participants were aged between 10.00 and 17.99 years with a body mass index (BMI) greater than the 90th percentile for age and sex [13]. Subjects were obese but otherwise healthy, free from psychiatric morbidity, and not taking any medications known to alter metabolism or body composition. The experimental protocol was approved by the Royal Children's Hospital, Brisbane (2006/096), and the University of Queensland Human Ethics Committee (2007000797). Written informed assent and consent were obtained by all adolescents and their parents prior to the commencement of the study.

### 2.1. Anthropometry

Height was measured to the last completed millimetre using a wall-mounted stadiometer (Holtain Instruments Limited Crymych, UK). Weight was measured to the nearest 0.05 kg using calibrated electronic scales (Tanita BWB-600 Wedderburn Scales, Australia). Body mass index (BMI) was calculated (weight (kg)/height (m^2^)) and *Z* scores (SD scores), for BMI, were calculated using the reference values for children from the Centers for Disease Control and Prevention [13]. Pubertal (Tanner [14]) stage was determined by a pediatrician.

### 2.2. Total Energy Expenditure

Total energy expenditure was measured using the DLW method as described in detail elsewhere [15]. A baseline urine sample was collected for the determination of the background isotope enrichment level. Subjects were then given an oral dose of DLW (^2^H_2_O and H_2_^18^O), approximately 0.083 g ^2^H_2_ (99.8 atom% excess; Sigma Aldrich, Milwaukee, WI) and 2.083 g ^18^O (10 atom% excess; Taiyo Nippon Sanso, Yokogawa, Japan) per kg of body water. Spot urine samples were collected after the dose at 5 hr and on ten consecutive days. Analysis of the isotopic enrichment was determined in duplicate with an Isoprime Dual Inlet Stable Isotope Ratio Mass Spectrometer (Isoprime Dual Inlet SIRM, MassLynx 4.0i Software, Isoprime, Manchester, UK) coupled in-line with a Multiprep-Gilson autosampler. Hydrogen analyses were done by a 3 hr equilibration with hydrogen gas at 40°C using Hokko coils. Oxygen analyses were done by a 10 hr equilibration with CO_2_ at 40°C. All samples were analyzed in duplicate and laboratory standards were calibrated using the international suite of waters SMOW, SLAP, and GISP. Results were reported in ‰ (delta per mil units) relative to SMOW. Total energy expenditure was calculated using the multipoint method. The ^2^H_2_ and ^18^O zero-time intercepts and elimination rates (kd and ko) were calculated using linear regression of the log of isotopic enrichments, relative to predose enrichment using postdose urine samples over the 10-day sample period. The zero-time intercepts were used to determine the dilution spaces at the time of the dose. Total body water (TBW) was then calculated using the ^2^H_2_ and ^18^O dilutions spaces (^2^H dilution space/1.04 and ^18^O dilution space/1.01, resp.). The production rates of carbon dioxide (rCO_2_) and water (rH_2_O) from the isotope elimination rates (kd and ko) and TBW were determined by the method of Wells et al. [16]. Oxygen consumption was then determined using the following equation:

(1) Oxygen consumption (l/min)  =carbon dioxide production (l/min⁡)0.85.

A respiratory quotient of 0.85 was used for each subject, as this value is indicative of the catabolism of a mixed diet over a period of days [17]. Using the production rate of carbon dioxide and the consumption rate of oxygen, TEE was then calculated using Weir's equation [17]. Consider

(2) TEE(kcal/24 hrs)  =3.941×O2  consumption  (l/day)   +1.106×CO2  production  (l/day).

### 2.3. Resting Energy Expenditure

Resting energy expenditure (REE) was measured via indirect calorimetry using a ventilated hood system with the subject resting supine after an overnight fast (Deltratrac II Metabolic Monitor, MBM-200, Datex-Deltatrac, Datex-Engstrom Division, Helsinki, Finland). The calorimeter was calibrated with a reference gas mixture of 95.00% O_2_ and 5.00% CO_2_. O_2_ consumption and CO_2_ production were measured at one minute intervals for 30 minutes and averaged over the whole measurement period. The subjects were required to rest in a supine position for 20 minutes prior to the commencement of the test. The first five to ten minutes were excluded from the analysis to account for environmental adjustment by the subjects and gas adaptation in the hood. REE was calculated from the measured oxygen consumption and carbon dioxide production similar to TEE according to the formula by Weir [17].

By measuring both TEE and REE, we therefore derived a measured activity energy expenditure (TEE − REE = AEE) or PAL values (PAL = TEE/REE).

### 2.4. Physical Activity

Physical activity was measured via a four-day self-reported diary, using a simplified version of activity dairies as described by Bouchard et al. [18], in conjunction with the measurement period of TEE using DLW. Diaries were completed on three week days and one weekend day [18]. Subjects were given verbal and written instructions with an example of how to complete the diary. Each day was divided into 96 × 15 minute intervals and the subjects were asked to record their activities on each day. On completion, these activities were categorised into nine levels according to their average energy costs, representing multiples of their respective metabolic equivalents (METs) assigned as per Ainsworth et al. [19, 20]. Total daily METs values were calculated and averaged to give a PAL value for each subject. At the end of the recording period, the diary was checked with a researcher for completeness and any clarification of recorded activities was sought from parents of the child participating. Compliance with completing the diary was 100% as subjects were required to return the diary at a scheduled appointment.

### 2.5. Statistical Analysis

Statistical computation was performed using the SPSS for Windows statistical package (Version 18.0; SPPS Inc, Chicago, IL). In order to evaluate the agreement between the two measurement instruments, Bland-Altman analysis was used [21]. Level of significance was set at 5% (*P* < 0.05) for all comparisons. Paired *t* tests were used to compare measured and estimated TEE and PAL values. Results are expressed as means ± SD.

## 3. Results

Twenty-two subjects, aged between 10.03 and 16.00 years, completed the study prior to entry into the Eat Smart program. All subjects were overweight with BMI *Z* scores ranging from 1.43 to 2.61. Summary characteristics are presented in Table 1. There were no statistically significant differences in any of the physical characteristics, TEE, or REE between boys and girls. Similarly, when TEE and REE were adjusted for FFM, using log-log regression, no significant gender differences were apparent.

**Figure 1:**
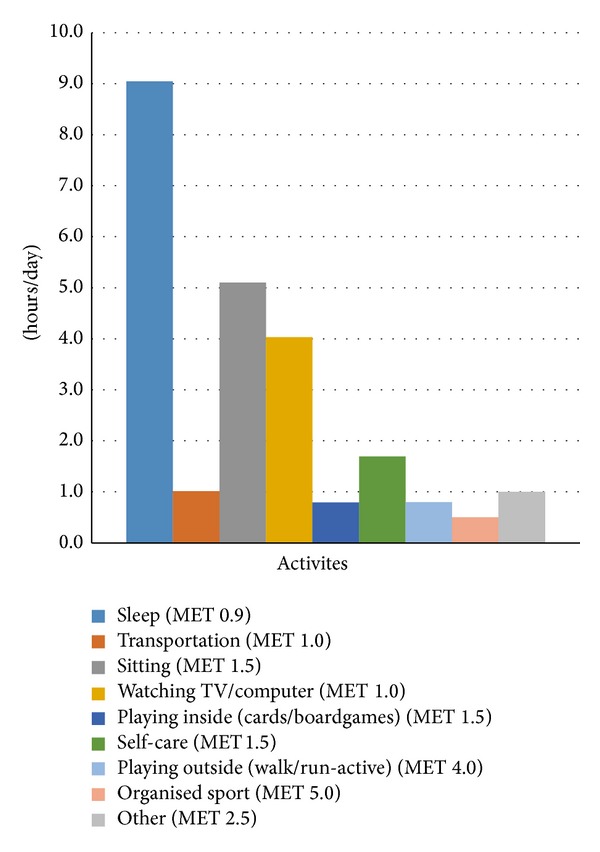
Time spent in physical activity estimated from physical activity diaries.

The proportion of energy used for physical activity (AEE) was, on average, 20% of their daily TEE. Time spent in sedentary activity (METs 1–1.5) occupied 45% of the day, while time spent in activity rated between 3.5 and 5.5 METs (i.e., playing outside and organised sport) occupied only 13% of the day.  Figure 1 shows the hours spent in each physical activity diary category per day.

**Figure 2:**
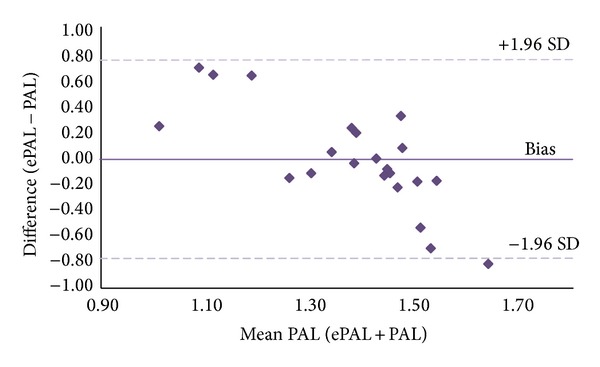
*Bland-Altman ePAL*. The Bland-Altman representation of the mean difference between estimated PAL values self-reported from physical activity diaries (ePAL) and measured PAL values obtained from measurements of TEE and REE (PAL = TEE/REE), in 22 obese children and adolescents.

The group as a whole were sedentary, with girls reporting on average 4.7 hours per day of “screen time” and boys 3 hours per day. At a group level, there was no statistically significant difference between mean measured and mean estimated PAL values (1.37 ± 0.13 versus 1.40 ± 0.34, resp., *P* = 0.74). A Bland-Altman analysis illustrated a significant relationship (*r* = −0.76, *P* ≤ 0.05) between the difference and the mean and between the two methods, demonstrating that the bias (−0.01 ± 0.39) was not consistent across the range of PAL values (Figure 2). Subjects with a low measured PAL tended to overreport their activity, while those with a higher measured PAL underreported their activity using the diary.

## 4. Discussion

Self-reported questionnaires are considered a useful tool to obtain qualitative data relating to habitual physical activity in children and adolescents. Physical activity diaries are often more economical and can provide information on the types and perceived intensity of activity not recorded from more objective measurement methods, such as accelerometers. While many physical activity diaries for the estimation of PAL values have been validated in healthy adults and children [22, 23], the use of these diaries to estimate PAL values in obese children and adolescents has not been previously reported.

In this present study, we compared estimated PAL values via self-reported diaries against energy expenditure measured via the reference DLW technique. Our findings show that obese adolescents tend to overestimate their PAL values when self-reporting their physical activity behaviours. At a group level, no statistically significant difference between measured and self-reported PAL values was observed. However, when determining the accuracy of these diaries to estimate PAL values using a Bland-Altman plot, correlation of significant magnitude was illustrated. Subjects with a low measured PAL tended to overreport their physical activity, following similar reporting patterns to obese adults [12].

Although in the presence of obesity the usefulness of self-reported measures of activity has been questioned, with obese adults shown to overreport their activity [24], many practitioners still utilise self-reported measures of activity to guide their choice of PAL value for the estimation of energy requirements.

The physical activity compendium developed by Ainsworth et al. [20], in 2002, was designed to facilitate the coding of physical activity diaries for the estimation of PAL values and provides activity codes and MET intensities for use in adults. Ridley and Olds [24] reported that using adult MET values was the most accurate assignment technique of energy costs to children's activities [25, 26], yet the energy cost of activity in obese children and adolescents may well be different. As mentioned previously, REE depends mainly on FFM, and as a result, in populations where FFM is altered, MET energy equivalents may vary significantly from the original kcal/kg/hour point of reference, as suggested by Ainsworth et al. [20].

A study by Ekelund et al. [27], investigating whether the intensity and duration of physical activity differed between obese and normal weight adolescents, has suggested that in obese children and adolescents the energy cost of any given physical activity is greater than in nonobese individuals. Moreover, Goran et al. [28] infer that perhaps the time spent in physical activity may be a more significant factor than energy expenditure attributable to physical activity, in the maintenance of energy balance.

The results from the present study, using a direct measure of physical activity, disagree with the findings of Goran et al. [28] and suggest that not only the amount of time spent participating in physical activity but also AEE might be associated with obesity and its development in children and adolescents. The mean PAL values for both boys (1.43) and girls (1.36) in the present study were much lower than the values previously reported in studies of children of similar ages [29–32]. In the current study, the mean values for AEE as a percentage of TEE (23.5% in males and 16.2% in females) were lower than those reported by Ekelund et al. [27] (38.7% in males and 36.3% in girls). The proportion of energy used for physical activity provided some insight as to whether physical activity, per se, could push the energy balance equation into deficit. The sample in this study used 20% of their daily energy expenditure for activity. In this sedentary population, a PAL value sufficient to push them into weight loss would require a substantial change in their activity. Schoeller [33] speculated that a relatively high PAL value of 1.75 is permissive for weight gain in a “society with unrestricted possibilities of excessive food consumption” [27, 33]. A recent study by Metcalf et al. [34] also implied that fatness leads to inactivity not the other way around. Currently, there is no consensus on whether energy intake or lack of energy expenditure is the driver for childhood obesity [35]. There is a need to develop a low cost, practical, and accurate measure of physical activity in obese children and adolescents. The accurate measurement of physical activity is critical for determining energy requirements, current levels of physical activity, monitoring compliance with physical activity guidelines, and understanding the dose-response relationship between physical activity and health.

One of the main limitations of this study was that data were captured at a single time point, when activity was objectively measured using DLW; hence, no data on reproducibility are available. Furthermore, the inference that subjects with a PAL value underreported their level of physical activity as demonstrated in Figure 2 needs to be interpreted with caution. While a significant trend was apparent as suggestive of the correlation between the mean and difference of the two methods, the group as a whole were very sedentary. Approximately 90% of subjects had a PAL value of less than 1.6. A “higher” observed PAL value may be because TEE was higher, due to the energy cost of activity being higher in obese, so whilst they do not think they are very active, the activity they do perform, cost more energy.

Conversely, given the poor performance of self-report, one would question the need of such investigation. The present study was also limited by its cross-sectional design. Thus, we cannot draw any conclusions as to whether an inactive lifestyle causes obesity or whether obesity leads to a physically inactive lifestyle. Additionally, the small sample size due mainly in part to the high cost of the 18-oxygen isotope when dosing large individuals meant that this was not a true validation study. However, one of the strengths of this study was that the major components of energy expenditure (TEE and REE) were measured using reference techniques; thus valid assessments of energy expended in physical activity could be accurately assessed.

## 5. Conclusion

Sedentary, obese adolescents tend to overestimate their activity levels or perhaps underreport the time spent in sedentary activity, such as screen time. In clinical practice, the misreporting of PAL values could have deleterious effects on any weight management programme, as energy requirements would be significantly overestimated or underestimated. It is therefore recommended that the use of a four-day self-reported physical activity diary for the estimation of a PAL value in obese children and adolescents is unreliable for this purpose.

Estimating the true energy cost of physical activity remains a challenge. It is therefore important that physical activity records are accurate for use in individuals, to assist in the prediction of energy requirements. Physical activity records have the unique advantage of providing additional information on the types of activity and time devoted by individuals to specific activities. However, as sedentary obese children and adolescents tend to overreport their PAL values, objective measurement instruments or techniques need to be developed, to be used either in place of, or in combination, with diaries.

## Figures and Tables

**Table 1:** Physical characteristics of all subjects.

	All (*n* 22) mean ± SD	Boys (*n* 11) mean ± SD	Girls (*n* 11) mean ± SD
Age (y.)	13.2 ± 1.8	13.4 ± 2.4	13.00 ± 1.5
Height (cm)	161.9 ± 12.1	162.5 ± 13.5	161.3 ± 11.1
Tanner stage (% 1, 2, 3, 4, and 5)	18, 9, 36, 9, 28	36, 0, 37, 18, 9	0, 18, 36, 0, 46
Weight (kg)	82.9 ± 20.5	82.8 ± 21.6	83.0 ± 20.5
BMI (kg/m^2^)	31.3 ± 4.6	31.0 ± 4.22	31.7 ± 5.1
BMI *Z* score	2.16 ± 0.33	2.18 ± 0.36	2.13 ± 0.30
REE (kcal/24 hr)	2042 ± 442	2078 ± 452	2004 ± 452
TEE (kcal/24 hr)	2846 ± 995	3018 ± 1313	2657 ± 531
AEE (kcal/24 hr)	519 ± 452	638 ± 729	387 ± 26
PAL	1.40 ± 0.34	1.43 ± 0.40	1.36 ± 0.27
ePAL	1.37 ± 0.13	1.39 ± 0.10	1.35 ± 0.45
Active behaviour (hrs)	3.0 ± 0.1	3.1 ± 0.1	3.0 ± 0.3
Sedentary behaviour (hrs)	10.7 ± 2.0	11.4 ± 2.3	10.1 ± 2.1

ePAL, PAL estimated by diary.

Active behaviour was defined as any activity in which the MET value ranged from 3.5 to 5.0, such as playing outside and sports. Sedentary behaviour was defined as any activity in which the MET values ranged from 1.0 to 1.5, such as going to school, transportation, and screen time.
